# Prenatal immune activation in mice induces long-term alterations in brain mitochondrial function

**DOI:** 10.1038/s41398-024-03010-x

**Published:** 2024-07-16

**Authors:** Edith M. Schneider Gasser, Ron Schaer, Flavia S. Mueller, Alexandra C. Bernhardt, Han-Yu Lin, Christian Arias-Reyes, Ulrike Weber-Stadlbauer

**Affiliations:** 1 Institute of Veterinary Pharmacology and Toxicology, Vetsuisse Faculty, University of Zurich, Zurich, 8057 Switzerland; 2 Institute of Veterinary Physiology, Vetsuisse Faculty, University of Zurich, Zurich, 8057 Switzerland; 3 Department of Pediatrics, Faculty of Medicine, Université Laval, Québec, QC Canada; 4 Neuroscience Center Zurich, University of Zurich, and ETH, Zurich, 8057 Switzerland

**Keywords:** Neuroscience, Pathogenesis

## Abstract

Prenatal exposure to infections is a risk factor for neurodevelopmental disorders in offspring, and alterations in mitochondrial function are discussed as a potential underlying factor. Here, using a mouse model of viral-like maternal immune activation (MIA) based on poly(I:C) (POL) treatment at gestational day (GD) 12, we show that adult offspring exhibit behavioral deficits, such as reduced levels of social interaction. In addition, we found increased nicotinamidadenindinucleotid (NADH)- and succinate-linked mitochondrial respiration and maximal electron transfer capacity in the prefrontal cortex (PFC) and in the amygdala (AMY) of males and females. The increase in respiratory capacity resulted from an increase in mitochondrial mass in neurons (as measured by complex IV activity and transcript expression), presumably to compensate for a reduction in mitochondrion-specific respiration. Moreover, in the PFC of control (CON) male offspring a higher excess capacity compared to females was observed, which was significantly reduced in the POL-exposed male offspring, and, along with a higher leak respiration, resulted in a lower mitochondrial coupling efficiency. Transcript expression of the uncoupling proteins (*UCP4* and *UCP5*) showed a reduction in the PFC of POL male mice, suggesting mitochondrial dysfunction. In addition, in the PFC of CON females, a higher expression of the antioxidant enzyme superoxide dismutase (SOD1) was observed, suggesting a higher antioxidant capacity as compared to males. Finally, transcripts analysis of genes involved in mitochondrial biogenesis and dynamics showed reduced expression of fission/fusion transcripts in PFC of POL offspring of both sexes. In conclusion, we show that MIA causes alterations in neuronal mitochondrial function and mass in the PFC and AMY of adult offspring with some effects differing between males and females.

## Introduction

Maternal immune activation (MIA) is an environmental risk factor causing alterations in the CNS of offspring and can lead to neurodevelopmental disorders (NDDs), including autism spectrum disorder (ASD), bipolar disorder, attention deficit hyperactivity disorder (ADHD), and schizophrenia [1–3].

Well-functioning mitochondria are essential for proper neuronal development and function. Specifically, mitochondrial function and dynamics play critical roles in neuronal development including neurite outgrowth and synaptogenesis [4, 5], and regulate the synaptic vesicle pool and spatial and working memory [6–8]. Reduced expression of mitochondrial electron transport system (ETS) genes has been documented in NDDs, including brain tissue from patients with ASD [9], and ADHD [10]. Moreover, recent evidence indicates that mitochondrial hypermetabolism precedes synaptic disorganization in animal models of NDD [11, 12]. We have recently reported that MIA induced in C57CBl6 mice by administration of the viral mimetic poly(I:C) (POL) at gestational day twelve (GD12) results in impaired social behavior and reduced expression of genes involved in oxidative phosphorylation in the prefrontal cortex (PFC) of male offspring [13]. However, it remains unknown whether MIA affects mitochondria and which cells, and brain areas are affected. Moreover, mitochondrial function shows marked sexual dimorphism in the brain, affecting steroid hormone synthesis, oxidative capacities, inflammatory responses, and reactive oxygen species (ROS) resistance [14]. While also NDDs are often sexually dimorphic with differences in genetics, affected brain areas, neuronal signaling, and behaviors [15**–**19], yet sex differences in brain mitochondrial function remain largely unexplored.

The aim of this study was to analyze the influence of MIA by a viral mimetic on mitochondrial (dys)function in cortical and subcortical brain regions of adult female and male offspring. Oxidative phosphorylation (OXPHOS), maximal electron transfer capacity (max ETC), and cytochrome C oxidase activity (complex IV, CIV) were evaluated using high-resolution respirometry [7, 20]. Cytochrome c oxidase activity, as a surrogate of mitochondrial content [7, 21], allowed the analysis of mitochondrion-specific respiration.

There is evidence for mitochondrial dysfunction and oxidative stress in brain tissue from individuals with ASD [22]. In addition, sex differences in biomarkers of oxidative stress in clinical studies of ASD are proposed [23]. Oxidative stress occurs when the concentration of ROS is elevated, and in this scenario, mitochondria leak protons across the inner mitochondrial membrane back into the matrix to reduce the production of ROS by mitochondrial protein complexes I and III. However, this process decreases the proton gradient and membrane potential, reducing the coupling efficiency between oxidation (electron passage through the ETS) and phosphorylation (ATP synthesis). In individuals with ASD, elevated respiratory rates in lymphoblastoid cells have been shown to be associated with more proton leak, uncoupling, and superoxide (a form of ROS) elevation in the mitochondrial compartment [24, 25]. Here, we evaluated the impact of MIA on the coupling efficiency, excess capacity of the ETS, and transcript expression of the brain uncoupling proteins UCP1, UCP2, UCP4, and UCP5 [26], and the antioxidant enzymes superoxide dismutase SOD1 and SOD2 isoforms as proxies for oxidative stress.

Failure in mitochondrial dynamics (fusion and fission) is also implicated in variations in mitochondrial respiration and was previously observed in individuals with ASD [27]. Here we assessed measures for mitochondrial biogenesis (peroxisome proliferator-activated receptor-gamma co-activator 1α (*PGC1α*)) and nuclear transcription factor 2 (*Nrf2*), mitochondrial pro-fusion mitofusin 1 (*Mfn1*) and mitofusin 2 (*Mfn2*), as well as pro-fission dynamin 1 *(Dnm1)* and dynamin 2 (*Dnm2*) were assessed via RT-qPCR.

Finally, to assess the cell specificity in mitochondrial alterations, levels of cytochrome C oxidase subunit 6a1 (*Cox6a1*), and cytochrome B (*CytB*) transcript expression were assessed with fluorescent in situ hybridization (RNAscope® Fluorescent Multiplex Assays) in neurons and microglia.

## Material and methods

### Animals

C57BL6/N mice, originally obtained from Charles River (Sulzfeld, Germany), were kept in our in-house specific pathogen-free facility until breeding began to generate poly(I:C) and control offspring (see below). All animal breeding and holding rooms were temperature- and humidity-controlled (21 ± 1 °C, 55 ± 5%) and kept under a reversed light-cycle (lights off: 9:00 A.M. to 9:00 P.M.). All animals had *ad libitum* access to food (Kliba 3436, Kaiseraugst, Switzerland) and water throughout the entire study. All procedures described in the present study were previously approved by the Cantonal Veterinarian’s Office of Zurich, Switzerland (License No. 124/2020) and all efforts were made to minimize the number of animals used and their suffering.

Experiments were performed in accordance with the official guidelines and regulations, following the ARRIVE guidelines [28].

### Timed mating

To obtain timed-pregnant dams for the subsequent maternal manipulations we used a timed-mating procedure as established and validated before [29]. Each mating cage contained 2 females and 1 male animal. Before mating, they were kept in a partitioned cage for 2 days, which allowed olfactory but not physical contact between male and female animals. On the third day of partitioning, female and male animals were brought together and allowed to mate. Successful mating was verified the next morning by the presence of a vaginal plug, and the day was referred to as GD0. Upon successful mating, the females were kept alone throughout the entire gestation and post-partum rearing periods.

### Maternal immune activation

Pregnant F0 dams on GD 12 were randomly assigned into two groups receiving either a single injection of poly(I:C) (potassium salt, P9582, lot number: 086M4045; Sigma-Aldrich, Buchs, St. Gallen, Switzerland) or vehicle. Poly(I:C) (5 mg/kg) was dissolved in sterile pyrogen-free 0.9% NaCl (vehicle) solution to yield a final concentration of 1 mg/ml and was administered intravenously (i.v.) into the tail vein under mild physical constraint as described before [29, 30]. The dose of poly(I:C) was selected based on our previous dose-response studies [31, 32].

A total of 2 cohorts were generated for the assessment of behavior and mitochondrial function (cohort 1, 14 dams) and RNAScope (cohort 2, 12 dams). In both cohorts, half of the animals were randomly assigned to poly(I:C) treatment (POL), and the other half to vehicle treatment (CON). The sample size was chosen based on earlier studies obtaining reliable data with the selected number of animals [32]. The selected gestational window (i.e., GD 12) was selected based on previous findings showing that poly(I:C) exposure at GD 12 leads to multiple behavioral abnormalities in the adult offspring, including reduced social behavior [13]. We have previously verified the effectiveness of this poly(I:C) lot and administration protocol in C57BL6/N mice in terms of the elicited cytokine-associated inflammatory response in maternal and fetal tissues [32, 33].

All offspring were weaned and sexed on postnatal day (PND) 21. Littermates of the same sex were caged separately and maintained in groups of 3 to 5 animals per cage. Upon reaching adulthood (PND 70 onwards) animals were subjected to a behavioral assessment of social interaction, followed by post-mortem molecular analyses after a resting phase of 14 days. For each cohort, 1–2 offspring per sex and litter were randomly selected and tested to minimize possible confounds arising from litter effects [34]. Both male and female offspring were used throughout the study.

### Behavioral testing

To confirm the effectiveness of the used MIA protocol resulting in behavioral abnormalities in males and females, we assessed social interaction (three-chamber social interaction test) in adult offspring [35]. This test is widely used and extensively validated previously in animal models of MIA [2, 36, 37], and routinely used in our laboratory [13, 31, 32].

**Fig. 1:**
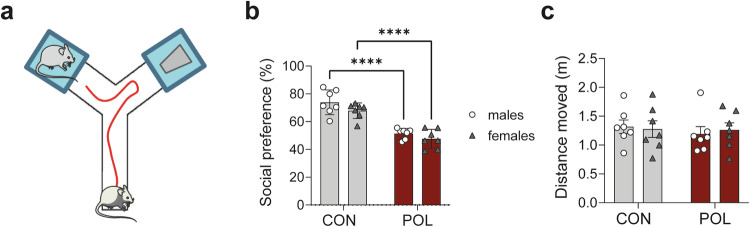
Social interaction of POL-exposed and control male and female adult offspring. **a** Social behavior test diagram. The relative exploration time between an unfamiliar congenic mouse and an inanimate dummy object is assessed. **b** Social interaction behavior in both sexes of the POL offspring compared to CON. Multiple comparisons: ****P < 0.0001. **c** Locomotor activity in both sexes of POL offspring compared to CON. Data points show the performance of individual animals (*n* = 7 per sex from 7 litter per group). Bar plots with individual values represent means ± SEM.

The **social interaction test** was performed in a Y-maze by analyzing the relative exploration time between an unfamiliar congenic mouse and an inanimate dummy object (Fig. 1a). Two out of the three arms contained a rectangular wire grid cage (13 cm × 8 cm × 10 cm). The third arm did not contain a metal wire cage and served as the start zone. One metal wire cage contained an unfamiliar C57BL6/N mouse of the same sex (10–12 weeks of age), whereas the other wire cage contained an inanimate dummy object. The latter was a black scrunchie made of velvet material. The allocation of the unfamiliar live mouse and inanimate dummy object to the two wire cages was counterbalanced across experimental groups. To start a test trial, the test mouse was gently placed in the start arm and allowed to explore freely for 5 min. Behavioral observations were made by an experimenter who was blinded to the experimental conditions, and social interaction is defined as nose contact within a 5-cm interaction zone. For each animal, the time spent with the unfamiliar mouse was calculated in percentage by the formula ([time spent with the mouse]/[time spent with the inanimate object + time spent with the mouse]) × 100. In addition to the analysis of social interaction, the total distance moved is recorded and analyzed to assess **locomotor activity**. This is achieved by a digital camera mounted above the Y maze, which captures images at a rate of 5 Hz. The images are transmitted to a PC running the EthoVision tracking system (Noldus Information Technology, Netherlands), which provides the measurement of the total distance moved (cm) during the 5-min testing period.

### Mouse brain collection for high-resolution respirometry and molecular analyses

Mice were killed by decapitation for subsequent respirometry, mRNA, and protein expression analyses. Brains were rapidly extracted from the skull (within <30 s) and micro punches of the medial PFC (mPFC) (including anterior cingulate, prelimbic, and dorsal parts of the infralimbic cortices; bregma: +2.3 to +1.3 mm) and the AMY (including major parts of the basolateral nuclei; bregma: −1.2 to −2.2 mm) regions were obtained. These brain regions have been previously shown to be affected in the MIA model [13, 29] and are of broad relevance to neurodevelopmental disorders [38**–**40].

Samples for high-resolution respirometry were blotted dry, assessed for wet weight in a balance-controlled scale (Dual Range Analytical Balance, Mettler Toledo AG, Switzerland), maintaining constant relative humidity and hydration consistency for the stability of measures, and immediately immersed in ice-cold respiration medium MiR05 (110 mM sucrose, 0.5 mM EGTA, 3 mM MgCl_2_*6H_2_O, 80 mM KCl, 60 mM K-lactobionate, 20 mM taurine, 10 mM KH_2_P0_4_, 20 mM HEPES, and 1 g/l bovine serum albumin, pH = 7.1) [7]. All chemicals were obtained from Sigma-Aldrich (Switzerland).

Samples for mRNA and protein analysis were snap-frozen in liquid nitrogen and stored at −80 °C until analysis.

### High-resolution respirometry

Mass-specific (pmol O_2_/s*mg tissue wet weight) oxygen consumption rates (OCR) were collected using a high-resolution Oxygraph-2k respirometer (Oroboros, Innsbruck, Austria). Standardized instrumental calibrations were performed to correct for the back-diffusion of oxygen into the chamber from various internal components, leak from the exterior, oxygen consumption by the chemical medium, and sensor oxygen consumption. All experiments were carried out in a hyper-oxygenated environment (>200 nmol/ml) to prevent any potential oxygen diffusion limitations and oxygen flux was resolved by software allowing nonlinear changes in the negative time derivative of the oxygen concentration signal (Dat Lab, Oroboros, Innsbruck, Austria). All measures were collected at 37 °C in a respiration buffer MiR06 (MiR05 + 280 IU/ml catalase) with saponin (50 μg/ml) to facilitate cell membrane permeabilization [41]. The basal mitochondrial respiration rate was evaluated following the substrate-uncoupler-inhibitor titration (SUIT) protocol as we described in ref. [7]. We analyzed various respiratory states representative of mitochondrial proton LEAK (L), maximal rates of coupled oxidative phosphorylation (P), and maximal rates of uncoupled electron transfer capacity (ETC). L-state respiration (N_L_), with kinetically saturating substrates (maleate (2 mM), pyruvate (5 mM), and glutamate (10 mM)) represents respiration due to proton leak across the inner mitochondrial membrane with a maximal proton motive force in the absence of ATP production and is comparable to the classical definition of either state 2 or 4 respiration [42]. P-state respiration with maximal NADH-linked electron input from mitochondrial complex I, N_P_, was induced following the addition of ADP (5 mM). Maximal rates of P-state respiration, and the best approximation to maximal oxidative phosphorylation potential in vivo, were then initiated with the addition of succinate (10 mM), which adds additional electron input from mitochondrial complex II, NS_P_. Maximal electron transfer capacity (max ETC, NS_E_ state) was then achieved with titrations of the protonophore carbonyl cyanide p-(trifluoromethoxy) phenylhydrazone (FCCP), in 0.5 μM steps up to an optimum concentration (ranging from 1.5 to 3 µM). E-state respiration primarily reflecting electron input via mitochondrial complex II is then determined following the addition of rotenone (0.5 µM) and consequent inhibition of mitochondrial complex I (S_E_).

Non-mitochondrial residual oxygen consumption (ROX) is measured with the addition of antimycin A (2.5 μM) and the attendant inhibition of mitochondrial complex III. Upon completing respiratory state analyses, ascorbate (2 mM) and N,N,N’,N’-tetramethyl-1,4-benzenediamine, dihydrochloride (TMPD, 0.5 μM) are simultaneously titrated into the chambers to assess cytochrome c oxidase (complex IV, CIV) activity via mass-specific OCR (pmol O_2_/s*mg ww). The chemical background oxygen flux due to autoxidation of TMPD in the presence of 2 mM ascorbate and a cytochrome c concentration of 10 μM was measured. Equation:

$${\rm{J}}^{\prime}_{{\rm O}2}={\rm{a}}^{\prime} +{\rm{b}}^{\prime} \,{{\rm{C}}}_{{\rm{O}}2}$$

was used as a basis for the chemical background correction (C_O2_, oxygen concentration (μM)).

Mitochondrion-specific respiration (%) was determined by normalizing mass-specific respiration (pmol O_2_/(s*mg ww)) to CIV activity (pmol O_2_/(s*mg ww)); (respiratory state respiration/CIV activity)*100.

Excess ETC capacity provides information on the coupling phosphorylation efficiency, and it is calculated as: max ETC (NS_E_)–max OXPHOS (NS_P_).

Physiological uncoupling or pathological decoupling are evident as an increased leak state in relation to ETC. Thus, coupling efficiency is calculated as: (max ETC (NS_E_)–Leak (N_L_)/ max ETC (NS_E_)).

### RNA isolation and real-time qPCR analyses

Total RNA was isolated using the SPLIT RNA extraction kit (Lexogen, Austria) following the manufacturer’s recommendations, and quantified by spectrophotometric analysis. RNA was analyzed by TaqMan RT-qPCR instrument (CFX384 real-time system, Bio-Rad Laboratories) using the iScript one-step RT-qPCR kit for probes (Bio-Rad Laboratories). The samples were run in 384-well formats in triplicates as multiplexed reactions with a normalizing internal control (36B4). We chose 36B4 as the internal standard for gene expression analyses since its expression was not affected by the prenatal treatments [43].

Thermal cycling was initiated with an incubation at 50 °C for 10 min (RNA retro-transcription) and then at 95 °C for 5 min (TaqMan polymerase activation). After this initial step, 39 cycles of PCR were performed. Each PCR cycle consisted of heating the samples at 95 °C for 10 s to enable the melting process and then for 30 s at 60 °C for the annealing and extension reaction. Relative target gene expression was calculated according to the 2(-Delta Delta C(T)) method [44]. Mouse TaqMan gene expression assays were used as summarized in Table 1.

**Table 1:** List of TaqMan gene expression assays used in this study.

Gene name	TaqMan assay ID
36b4	Custom-designed
Autophagy related 5 (ATG5)	Mm01187303_m1
Dynamin (DNM) 1	Mm00802468_m1
Dynamin (DNM) 2	Mm00514582_m1
Mitofusin (MFN) 2	Mm00500120_m1
Mitofusin (MFN) 1	Mm00612599_m1
NADPH oxidase 2 (NOX2)	Mm01287743_m1
Nuclear factor erythroid 2-related factor 2 (NRF2)	Mm00477784_m1
Peroxisome proliferator-activated receptor gamma coactivator 1-alpha (PGC1a)	Mm01208835_m1
Superoxide dismutase (SOD) 1	Mm01344233_g1
Superoxide dismutase (SOD) 2	Mm01313000_m1
Uncoupling protein (UCP) 1	Mm01244861_m1
Uncoupling protein (UCP) 2	Mm00627599_m1
Uncoupling protein (UCP) 4	Mm00511820_m1
Uncoupling protein (UCP) 5	Mm00488302_m1

The list summarizes the names of the genes of interest and their TaqMan assay ID, listed according to alphabetical order. 36b4 was custom-designed, with the following probe and primer sequences:

36b4 forward primer: 5′-AGATGCAGCAGATCCGCAT-3′; reverse primer: 5′-GTTCTTGCCCATCAGCACC-3′; probe: 5′-CGCTCCGAGGGAAGGCCG-3′.

### RNA scope

To assess cell specificity of mitochondrial gene expression, we performed fluorescent multiplex in situ hybridization (RNAscope Multiplex Fluorescent v2 Assay; Advanced Cell Diagnostics) in a new cohort of animals. Following rapid decapitation of mice, brains were rapidly frozen in powdered dry ice and stored at −80 °C. Coronal sections of OCT-embedded brains were cut at 20 μM at −20 °C and thaw-mounted onto Super Frost Plus slides (Fisher). Slides were stored at −80 °C until further processing. Fluorescent in situ multiplex hybridization was performed according to the RNAscope v2.0 Fluorescent Multiple Kit User Manual for fresh frozen tissue (Advanced Cell Diagnostics, Inc., CA, USA). Briefly, sections were fixed in 4% PFA, dehydrated with increasing percentage of EtOH, and treated with pretreatment-4 protease solution (Advanced Cell Diagnostics, Inc., CA, USA). Sections were then incubated with target probes for mouse NeuN to label neurons (RNAscope® Probe - Mm-Rbfox3, Cat No. 313311, accession number NM_001039167.1, probe region 1827–3068), mouse Iba-1 to label microglia (RNAscope® Probe—Mm-Aif1, Cat No. 319141, accession number NM_019467.2, probe region 31–866), and probes for Cox6a1 and Cytochrome B to assess mitochondrial markers (RNAscope® Probe—Mm-Cox6a1, Cat No. 519781, accession number NM_007748.3, probe region 2–510; RNAscope® Probe—Mm-mt-Cytb, Cat No. 517301, accession number NM_ 005089.1, probe region 14146–15270). Following probe hybridization, sections underwent a series of probe signal amplification steps followed by incubation of fluorescently labeled probes (Opal fluorophore reagent pack, PerkinElmer, MA, USA) designed to target the specified channel associated with the genes of interest (mt-cytb-C1, Mm-Aif1-C2, Mm-Cox6a1-C3, Mm-Rbfox3-C4). Slides were counterstained with DAPI, and coverslipped with fluorescent mounting medium (Dako, Agilent Technologies, CA, USA). High-resolution images were obtained on a fluorescent microscope (20×, Akoya PhenoImager™ HT, Perkin Elmer), and analyzed using QuPath.

The mRNA levels of CytB and Cox6a1-positive neurons and microglia were quantified using QuPath [45] by a blinded experimenter by quantifying expression levels in NeuN- or Iba-1 only positive cells. The analysis involved the manual delineation of analysis areas in medial prefrontal cortex and the amygdala, spanning three consecutive sections from anterior to posterior direction. Within the region of interest, cell nuclei and estimated cytoplasm were identified using DAPI signals. For each section, a minimum of 50 single cells were analyzed. To distinguish microglia and neurons, the threshold for Iba-1+ cell (representing microglia) and NeuN+ cell (representing neurons) was established using the “object classification” tool. These thresholds were kept constant during the image analysis process. The mean intensity of Cytb and Cox6a1 was measured within Iba-1+ cells for microglia and NeuN+ cells for neurons, respectively. The results were displayed as the mean intensity of Cytb and Cox6a1 within microglia and neurons.

### Statistical analyses

All data were analyzed by two- (treatment and sex) or three (treatment, sex, and mitochondrial states)-way analyses of variance (ANOVA) followed by Tukey’s or Fisher’s post-hoc multiple comparisons whenever appropriate. The F value is given for each result. Statistical analyses were performed using GraphPad Prism (version 9.0.0 for Windows, GraphPad Software, Boston, MA, USA, www.graphpad.com), and statistical significance was set at *P* < 0.05.

## Results

To confirm the effectiveness of the MIA protocol in our cohorts, male and female adult (12 weeks onwards) POL and CON offspring were subjected to a social behavioral testing (Fig. 1). We used the three-chamber social interaction test [34] by analyzing the relative exploration time between an unfamiliar congenic mouse and an inanimate dummy object (Fig. 1a). Animals selected for the analysis in mitochondrial function had a deficit in social behavior similar in both sexes (2-way ANOVA, *F*_(1,24)_ = 75.69, *P* < 0.0001, Fig. 1b). Social impairment was not confounded by alterations in basal locomotor activity (Fig. 1c). Thus, the chosen poly(I:C) prenatal immune activation regime induced the expected long-term social behavioral alterations.

### Increased oxidative phosphorylation (OXPHOS)-linked respiration in the prefrontal cortex (PFC) and the amygdala (AMY) of POL-exposed adult offspring mice

**Fig. 2:**
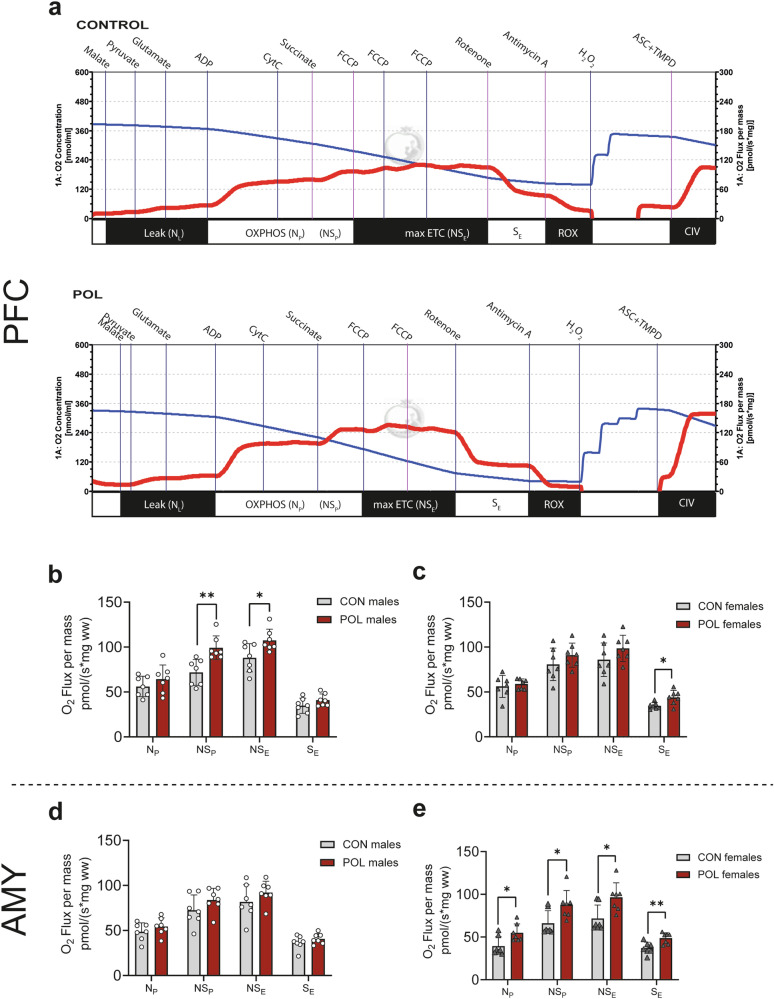
Oxidative phosphorylation (OXPHOS)-linked respiration in the PFC and AMY from POL-exposed and control male and female adult offspring. **a** Representative respirometry traces from a control (CON) (top panel) and a poly(I:C) (POL) (bottom panel) prefrontal cortex (PFC) tissue, which illustrates the change in oxygen concentration (nmol/ml, left *y*-axis, blue line) and oxygen flux per mass (pmol O_2_/(s*mg ww, right *y*-axis, red line)) in adult male mice. Respiratory states were achieved following the SUIT protocol (Substrate, Uncoupling, Inhibition, Titration), consisting of (left-to-right): LEAK (N_L_) without adenylates (maleate, pyruvate, glutamate); coupled respiration with maximal electron input specific to mitochondrial complex I (N_P_; addition of ADP); maximal electron input specific to mitochondrial complex I and II (NS_P_; addition of succinate); maximal uncoupled respiration with electron input from complexes I and II (NS_E_, step addition of carbonyl cyanide p-trifluoromethoxy phenylhydrazone (FCCP) until maximal increase); uncoupled respiration with maximal electron input specific to mitochondrial complex II (S_E_, addition of rotenone); and non-mitochondrial residual oxygen consumption (ROX; addition of antimycin A). Following respiratory state analysis, peroxide (H_2_O_2_) was added to reoxygenate the chambers to 350 nmol/ml O_2_ and ascorbate (ASC) and *N*′, *N*′, *N*′, *N*′-tetramethyl-1,4-phenylendiamine, (TMPD) were simultaneously added to assess cytochrome *c* oxidase (complex IV, CIV) activity. Mass-specific OXPHOS coupled respiration (N_P_, NS_P_), maximal ETC (NS_E_), and maximal CII-respiration (S_E_) in: (**b**) PFC of male mice offspring. Multiple comparisons: **P = 0.003 and *P = 0.026, (**c**) PFC of female mice offspring. Multiple comparisons: *P = 0.02, (**d**) AMY of male mice offspring, and (**e**) AMY of female mice offspring. Multiple comparisons: *P ≤ 0.02, **P = 0.0067. Bar plots with individual values represent means ± SD. *n* = 7 per sex from 7 litter per group.

We used high-resolution respirometry to assess mitochondrial respiratory control in PFC and AMY tissue from both POL-exposed male and female adult and control offspring. Mitochondrial respiratory traces collected from the PFC in control (top) and POL (bottom) males are illustrated in Fig. 2a. Mass-specific leak (N_L_), NADH-linked respiration (N_P_), NADH- and succinate-linked respiration (NS_P_), non-coupled maximal electron transfer capacity (NS_E_), non-coupled succinate-linked respiration (S_E_), residual non-mitochondrial oxygen consumption (ROX); and cytochrome C activity (complex IV) were evaluated using a respirometry SUIT protocol as described before [7]. A significant increase in the different mitochondrial states was observed in the PFC of the POL-exposed offspring mice (3-way ANOVA, *F*_(3,72)_ = 6.5, *P* = 0.0006, Fig. 2b, c). In males, a significant increase in NS_P_ and NS_E_ was observed (2-way ANOVA, *F*_(1,48)_ = 20.6, *P* < 0.001, Fig. 2b). In females, a significant increase in S_E_ was observed (2-way ANOVA, *F*_(1,48)_ = 6.5, *P* = 0.013, Fig. 2c). Also in the AMY a significant increase in the mitochondrial states was observed in POL offspring compared to controls (3-way ANOVA, *F*_(3,72)_ = 3.3, *P* = 0.024). In POL males, comparison of individual respiration states was not significantly different (2-way ANOVA, *F*_(1,48)_ = 5.6, *P* = 0.022, Fig. 2d). On the contrary, in females all states were significantly increased (2-way ANOVA, *F*_(1,48)_ = 28.15, *P* < 0.0001, Fig. 2e). Thus, sex-specific differences in mitochondrial function in the PFC and the AMY are observed in offspring after MIA, with males showing a higher mitochondrial respiration in the PFC and females in the AMY.

### Increased mitochondrial content in the PFC and AMY from POL-exposed adult offspring mice compensates for a reduction in mitochondrial function

**Fig. 3:**
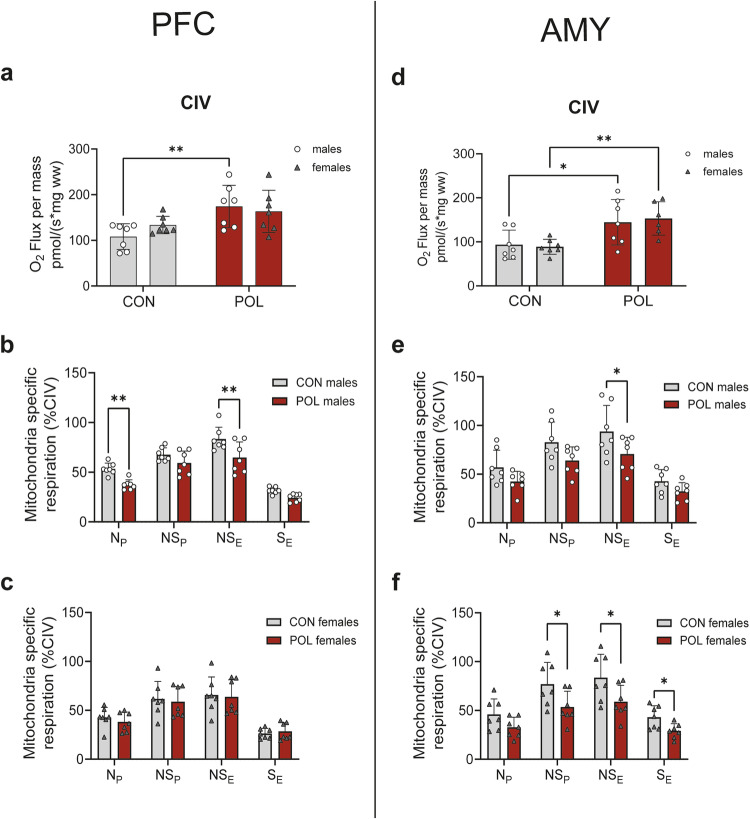
Cytochrome *c* oxidase (CIV) activity and mitochondrion-specific respiration in the PFC and AMY from POL-exposed and control male and female adult offspring. **a** Cytochrome *c* oxidase activity (CIV, pmol O_2_ /(s*mg ww)) in the PFC of male and female adult offspring. POL-exposed mice offspring show higher CIV activity. Multiple comparisons: **P = 0.0052. **b** Mitochondrion-specific respiration for N_P_, NS_P_, NS_E_, and S_E_ in the PFC of male offspring. Multiple comparisons: **P = 0.003. **c** Mitochondrion-specific respiration for N_P_, NS_P_, NS_E_, and S_E_ in the PFC of female offspring. **d** Cytochrome *c* oxidase activity (CIV, pmol O_2_/(s*mg ww)) in the AMY of male and female adult offspring. Multiple comparisons: *P < 0.05, **P = 0.0036. **e** Mitochondrion-specific respiration for N_P_, NS_P_, NS_E_, and S_E_ in the AMY of male offspring. Multiple comparisons: *P = 0.05. **f** Mitochondrion-specific respiration for N_P_, NS_P_, NS_E_, and S_E_ in the AMY of female offspring. Multiple comparisons: *P ≤ 0.05. Bar plots with individual values represent means ± SD. *n* = 7 per sex from 7 litter per group.

Cytochrome *c* oxidase (complex IV, CIV) activity, validated as a surrogate of mitochondrial content, was higher in the PFC of males (2-way ANOVA, *F*_(1,24)_ = 11.85, *P* = 0.002; Fig. 3a) and in the AMY of both sexes (2-way ANOVA, *F*_(1,24)_ = 18.7, *P* = 0.0002, Fig. 3d) in POL-exposed adult offspring mice compared to CON. Mitochondrion-specific respiratory analysis (mass-specific respiration normalized to a surrogate of mitochondrial content, e.g., CIV activity) helps distinguish qualitative alterations in respiratory control from quantitative differences driven primarily by changes in mitochondrial content. When normalizing mass-specific OXPHOS respiration to CIV, MIA caused a significant decrease in mitochondrial respiration in the PFC of male offspring (3-way ANOVA, *F*_(3,36)_ = 3.4, *P* = 0.028) (Fig. 3b) but not in females (Fig. 3c). On the contrary, in the AMY of POL-exposed adult offspring a significant reduction in mitochondrial respiration states was observed in both sexes (3-way ANOVA, *F*_(3,72)_ = 4.8, *P* = 0.004, Fig. 3e, f). Our data suggest that the mass-specific increase in mitochondrial respiration observed in the PFC in males and in the AMY of males and females POL-exposed adult offspring mice is attributable to quantitative increases in mitochondrial content to compensate for a reduction in mitochondrial function.

### Reduced excess capacity, coupling efficiency, and transcript expression of uncoupling proteins UCP4 and UCP5 were observed in the PFC of POL-exposed male offspring

Next, we evaluated the impact of MIA on the excess capacity and coupling efficiency of the ETS, and on the transcript expression of the brain uncoupling proteins UCP1, UCP2, UCP4 and UCP5 and the antioxidant enzymes superoxide dismutase (SOD)1 and SOD2.

**Fig. 4:**
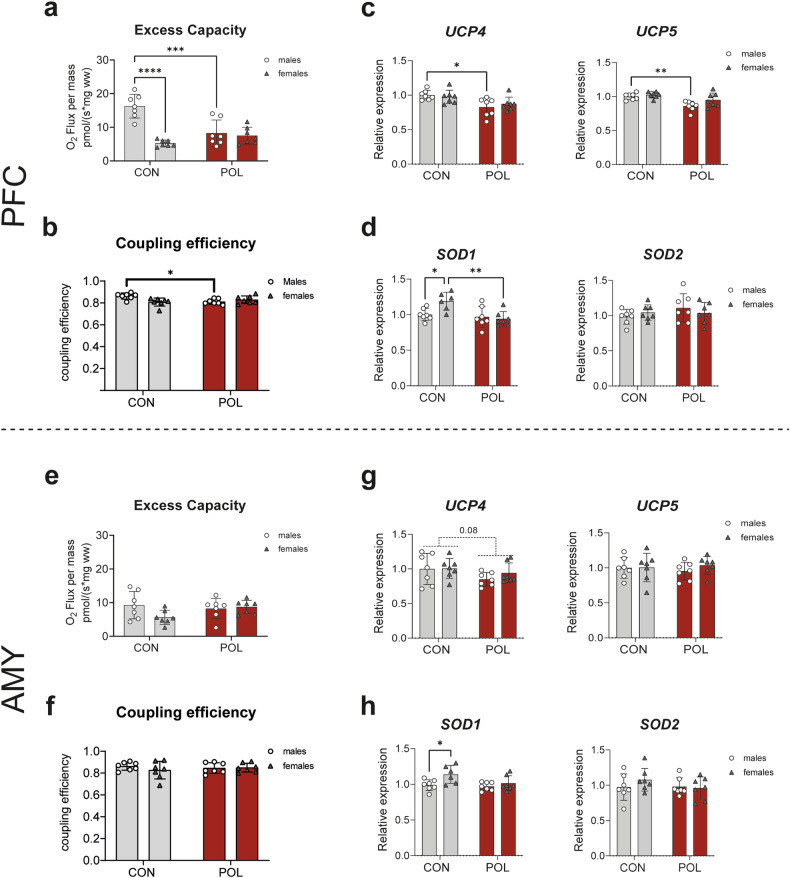
Excess capacity, coupling eficiency, and transcript expression of uncoupling proteins (UCP)s and antioxidant enzymes superoxide dismutase (SODs) in PFC and AMY from POL-exposed and control male and female offspring. **a** Excess ETC in the PFC. A significantly higher excess ETC is observed in control males than in control females and POL-exposed mice. Multiple comparisons: ****P < 0.0001 and ***P = 0.0002. **b** Coupling efficiency in the PFC. A reduction in coupling efficiency is observed in POL-exposed males. Multiple comparisons: *P = 0.03. **c** Transcript expression analysis of the uncoupling proteins 4 (*UCP4*) and 5 (*UCP5*) in the PFC with significantly less transcripts in the POL-exposed offspring. Multiple comparisons: *UCP4* *P = 0.026, and *UPC5* **P = 0.0066. **d** Transcript expression analysis of the antioxidant enzymes superoxide dismutase 1 (*SOD1)* and 2 (*SOD2*) in the PFC with significantly higher *SOD1* expression in control females than in control males and POL-exposed offspring. Multiple comparisons: *P = 0.049, **P = 0.0058. **e** Excess ETC in the AMY. No significant differences in excess ETC are detected across experimental groups. **f** Coupling efficiency in the AMY. No alterations were observed. **g** Transcript expression analysis of UCPs in the AMY. POL-exposed male and female offspring mice show an apparent (but non-statistically significant) reduction in *UCP4* and no alterations in *UPC5*. **h** Transcript expression analysis of *SODs* in the AMY. Higher *SOD1* expression in control females than in control males is observed. Multiple comparisons: *P = 0.046. Bar plots with individual values represent means ± SD. *n* = 7 per sex from 7 litter per group.

In the PFC of control males, a greater electron transfer excess capacity than in females was observed (2-way ANOVA, *F*_(1,24)_ = 6.56, *P* = 0.0171, Fig. 4a), reflecting that mitochondria in females function at a rate closer to their maximum capacity than in males. After MIA, the excess capacity of males is significantly reduced reaching similar levels to females (2-way ANOVA, *F*_(1,24)_ = 28.05, *P* < 0.0001, Fig. 4a). Moreover, a reduction in coupling efficiency, which can lead to increased production of ROS and uncoupling, is observed in males (2-way ANOVA, *F*_(1,24)_ = 4.94, *P* = 0.036, Fig. 4b). Transcription analysis of uncoupling proteins (UCP)s 1, 2, 4 and 5, showed a reduction in the expression of *UCP4* (2-way ANOVA, *F*_(1,24)_ = 12.49, *P* = 0.0017, Fig. 4c left panel) and *UCP5* (2-way ANOVA, *F*_(1,24)_ = 16.07, *P* = 0.0005, Fig. 4c right panel) in the PFC of POL-exposed males, without alteration in the expression of *UCP1* (2-way ANOVA, *F*_(1,24)_ = 1.8, *P* = 0.1922, Supplementary Fig. 1a) and *UCP2* (2-way ANOVA, *F*_(1,24)_ = 0.76, *P* = 0.39, Supplementary Fig. 1b) in both sexes, therefore the reduction in coupling efficiency does likely not result from increased uncoupling. In addition, lower transcript expression of the antioxidant enzyme superoxide dismutase (*SOD1*) was observed in the PFC of control males compared to females (2-way ANOVA, *F*_(1,23)_ = 10.48, *P* = 0.0053, Fig. 4d, left panel) suggesting lower response to oxidative stress. MIA caused no alterations in *SOD2* expression (2-way ANOVA, *F*_(1,24)_ = 1.15, *P* = 0.29, Fig. 4d, right panel). Together, the decrease in mitochondrial coupling efficiency in PFC of POL-exposed males without increase in uncoupling and a reduced transcript expression of SOD1, suggest a higher production of ROS than in females.

In the AMY, no alterations in excess capacity (*F*_(1,24)_ = 2.008, *P* = 0.169, Fig. 4e), and coupling efficiency (2-way ANOVA, *F*_(1,24)_ = 0.62, *P* = 0.44, Fig. 4f) were observed in POL-exposed offspring compared to controls. An apparent (but not-statistically significant) reduction in *UCP4* after MIA was observed in both sexes (2-way ANOVA, *F*_(1,24)_ = 3.15, *P* = 0.08, Fig. 4g left panel) with no alterations in *UCP5* (2-way ANOVA, *F*_(1,24)_ = 0.60, *P* = 0.44, Fig. 4g, right panel), *UCP1* (2-way ANOVA, *F*_(1,24)_ = 0.7, *P* = 0.4, Supplementary Fig. 1c), and *UCP2* (2-way ANOVA, *F*_(1,24)_ = 3.7, *P* = 0.07, Supplementary Fig. 1d). Lower transcript expression of *SOD1* was observed in AMY of control males compared to females (2-way ANOVA, *F*_(1,23)_ = 7.44, *P* = 0.01, Fig. 4h, left panel) with no alterations in *SOD2* expression in either sex of POL-exposed offspring (2-way ANOVA, *F*_(1,24)_ = 0.46, *P* = 0.5, Fig. 4h, right panel).

Together, our results thus indicate a higher production and vulnerability for ROS in the PFC of POL-exposed male offspring.

### Expression of fission/fusion mitochondrial dynamics transcripts is reduced in the PFC of POL-exposed offspring

**Fig. 5:**
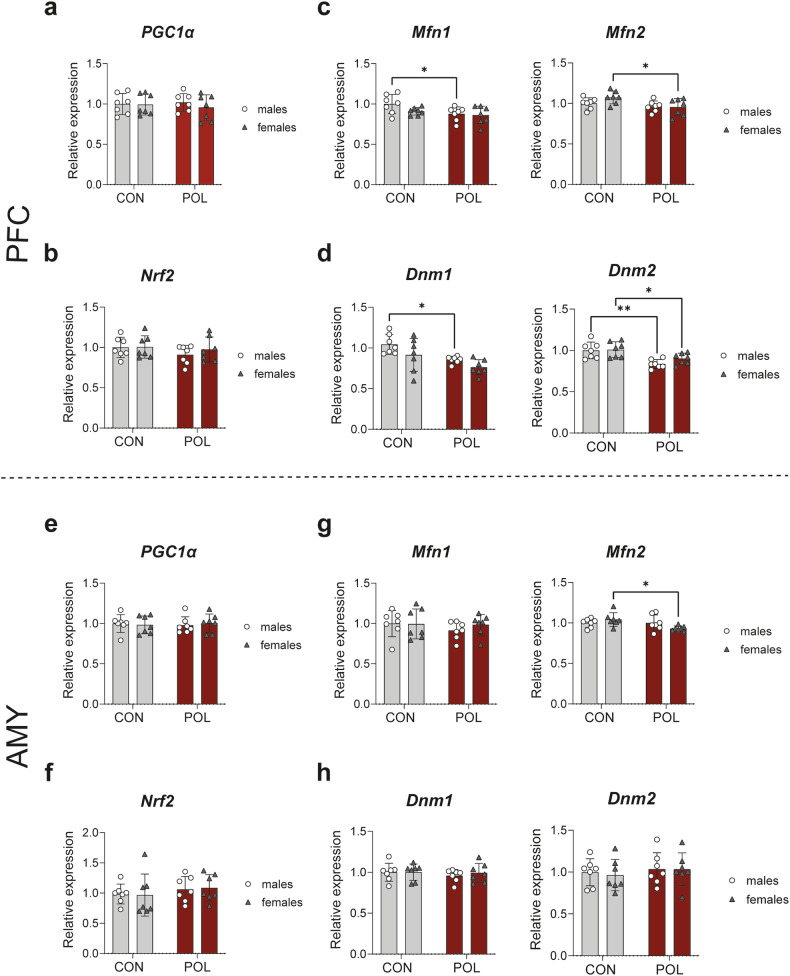
Transcriptional analysis of mitochondria biogenesis in PFC and AMY of control and POL-exposed male and female offspring. **a** *PGC-1α* mRNA expression in the PFC. There is no alteration across groups. **b** *Nrf2* mRNA expression in the PFC. There is no alteration across groups. **c** Pro-fusion: mitofusin 1 (*Mfn1*) and mitofusin 2 (*Mfn2*) mRNA expression in the PFC. Reduced expression in POL-exposed offspring mice. Multiple comparisons: *Mfn1*, *P = 0.044 and *Mfn2*, *P = 0.023. **d** Pro-fission: dynamin 1 (*DRP1*/*Dnm1*) and dynamin 2 (*Dnm2*) mRNA expression in the PFC. Reduced expression in POL-exposed offspring mice. Multiple comparisons: Dnm1, *P = 0.02 and Dnm2, *P = 0.02, **P = 0.002. **e** *PGC-1α* mRNA expression in the AMY. There is no alteration across groups. **f** *Nrf2* mRNA expression in the AMY. There is no alteration across groups. **g** Pro-fusion: *Mfn1* and *Mfn2* mRNA expression in the AMY. There is no alteration across groups in *Mfn1* but a reduction in *Mfn2* in POL-exposed females. Multiple comparisons: *P = 0.01. **h** Pro-fission: *Dnm1* and *Dnm2* mRNA expression in the AMY. There is no alteration across groups. Bar plots with individual values represent means ± SD. *n* = 7 per sex from 7 litter per group.

Transcriptional analysis of pathways regulating mitochondrial biogenesis was performed in PFC and AMY of POL-exposed male and female offspring and controls. No alteration in the transcript expression of peroxisome proliferator-activated receptor-γ coactivator 1-a (*PGC1α*) in the PFC (2-way ANOVA, *F*_(1,24)_ = 0.027, *P* = 0.87, Fig. 5a) and the AMY (2-way ANOVA, *F*_(1,24)_ = 0.00, *P* = 0.99, Fig. 5e) was observed across groups, nor in the transcript expression of the nuclear factor erythroid 2 (NFE2)-related factor 2 (*Nrf2*) in the PFC (2-way ANOVA, *F*_(1,24)_ = 1.43, *P* = 0.24, Fig. 5b) and the AMY (2-way ANOVA, *F*_(1,24)_ = 1.15, *P* = 0.29, Fig. 5f). Alterations in fission/fusion mitochondrial dynamics transcripts were observed in the PFC of POL-exposed offspring of both sexes, with a reduction in expression in the mitochondrial fusion transcripts mitofusin 1 (*Mfn1*) (2-way ANOVA, *F*_(1,24)_ = 5.58, *P* = 0.026, Fig. 5c left panel), and mitofusin 2 (*Mfn2*) (2-way ANOVA, *F*_(1,24)_ = 6.29, *P* = 0.019, Fig. 5c, right panel); and a reduction in expression in the mitochondrial fission transcripts dynamin 1 (*Dnm1*) (2-way ANOVA, *F*_(1,24)_ = 12.24, *P* = 0.002, Fig. 5d left panel) and dynamin 2 (*Dnm2*) (2-way ANOVA, *F*_(1,24)_ = 19.67, *P* = 0.0002, Fig. 5d right panel). In the AMY from POL-exposed offspring, only a statistically significant alteration in *Mfn2* transcripts (2-way ANOVA, *F*_(1,24)_ = 4.47, *P* = 0.045, Fig. 5g right panel) was observed, with no alterations in *Mfn1* (2-way ANOVA, *F*_(1,24)_ = 0.69, *P* = 0.41, Fig. 5g left panel), and the fission transcripts *Dnm1* (2-way ANOVA, *F*_(1,24)_ = 0.41, *P* = 0.52, Fig. 5h left panel), and *Dnm2* (2-way ANOVA, *F*_(1,24)_ = 0.59, *P* = 0.45, Fig. 5h right panel). These data suggest that MIA downregulates transcriptional signaling for mitochondrial pro-fusion and pro-fission dynamics mostly in the PFC of male and female offspring.

### Increase in cytochrome B (*CytB*) and cytochrome C oxidase (COX) transcripts in neurons in the PFC and AMY from POL-exposed male and female offspring

**Fig. 6:**
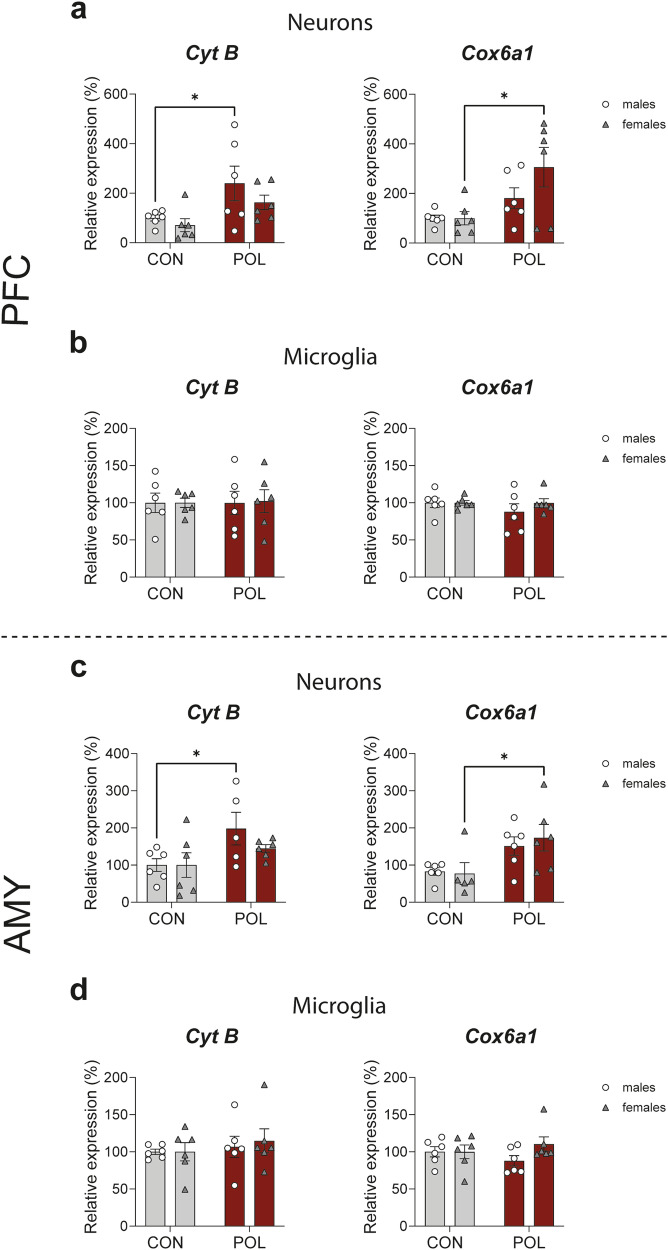
RNA scope in situ hybridization of cytochrome B (*Cyt B*) and cytochrome c oxidase subunit 6a1 (*Cox6a1*) in neurons and microglia of PFC and AMY from control and POL-exposed male and female offspring. **a** Neuronal *CytB* and *Cox6a1* mRNA expression in the PFC. Transcriptional expression is increased in neurons from POL-exposed offspring. Multiple comparisons: *CytB*: *P = 0.049, Cox6a1: *P = 0.012. **b** Microglia *CytB* and *Cox6a1* mRNA expression in the PFC. Transcriptional expression is unaltered. **c** Neuronal *CytB* and *Cox6a1* mRNA expression in the AMY. Transcriptional expression is increased in neurons from POL-exposed offspring. Multiple comparisons: *CytB*: *P = 0.049, Cox6a1: *P = 0.04. **d** Microglia *CytB* and *Cox6a1* mRNA expression in the AMY. Transcriptional expression is unaltered. Bar plots with individual values represent means ± SEM. *n* = 6 per sex from 6 litter per group.

Cell-specific expression of cytochrome B (*CytB*) and cytochrome C oxidase subunit 6a1 (*Cox6a1*) was determined by fluorescence in situ hybridization analysis (fISH), using target probes for specifically labeling neurons (*NeuN*) and microglia (*Iba-1*) (Supplementary Fig. 2). *CytB* and *Cox6a1* transcript expression was increased in POL-exposed male and female offspring respectively, in neurons (*NeuN*) in the PFC (2-way ANOVA: *CytB*, *F*_(1,20)_ = 8.11, *P* = 0.0099 and *Cox6a1*, *F*_(1,20)_ = 9.2, *P* = 0.006, Fig. 6a), and in the AMY (2-way ANOVA, *CytB*, *F*_(1,19)_ = 6.6, *P* = 0.018 and *Cox6a1*, *F*_(1,19)_ = 9.6, *P* = 0.006, Fig. 6c). No changes were observed in transcript expression in microglia (*Iba-1*) in the PFC (2-way ANOVA, *CytB*, *F*_(1,20)_ = 0.01, *P* = 0.94 and *Cox6a1*, *F*_(1,20)_ = 0.77, *P* = 0.39, Fig. 6b) and in the AMY (2-way ANOVA, *CytB*, *F*_(1,20)_ = 0.7, *P* = 0.40 and *Cox6a1*, *F*_(1,20)_ = 0.01, *P* = 0.19, Fig. 6d).

In conclusion, we here show that MIA causes alterations in mitochondrial mass and function, and transcript expression in neurons of the PFC and the AMY of male and female offspring.

## Discussion

We had previously shown that POL-induced MIA in mice is related to behavioral and molecular alterations with relevance to psychiatric-like disorders including schizophrenia and autism in the offspring [2]. While the long-term behavioral consequences of MIA are well-established, the underlying molecular and metabolic mechanisms remain poorly understood. Recently, we have identified that MIA leads to a reduction in many OXPHOS-related genes expression in the medial PFC of adult male offspring [13]. Interestingly many of these genes were also demonstrated to be markedly deregulated in NDDs, such as schizophrenia [46, 47]. Although it was found that MIA can induce changes in mitochondrial function in the brain of offspring [48], it remained largely unknown whether MIA induces lasting changes in brain mitochondrial function in adult offspring. In this study, we provide evidence that MIA increases mitochondrial function in the PFC and AMY in adult offspring of both sexes. This mitochondrial hyperactivity seems to result from an increase in mitochondrial mass to overcompensate for an apparent mitochondrial dysfunction. Measurements of the metabolic transcripts Cyt B and COX6a1, as markers of mitochondrial content, in neurons and microglia showed an increase only in neurons, hence indicating that MIA at GD12 significantly impacts neuronal mitochondrial function and metabolism in the adult offspring. Evidence for mitochondrial hyperactivity and altered social behavior was previously causally linked to a reduction in synaptic release [11, 12]. In line with this notion, earlier work from us demonstrated that enhanced mitochondrial neuronal respiration during postnatal development leads to higher vesicle number in presynaptic terminals [7] and enhanced synapse transmission [49]. Thus, although this work does not address alterations in synaptogenesis or synaptic function, it seems plausible that hyperactive neuronal mitochondria after MIA may lead to GABAergic synaptic alterations in mPFC and AMY, thereby causing social and emotional behavioral alterations. Indeed, increasing evidence indicates mitochondrial dysfunction in a range of NDDs including ASD [5, 50–52]. While these disorders are associated with several sex-dependent effects [53, 54], sex dimorphism and etiology and the underlying mechanisms are poorly understood. Consequently, sex-specific treatments are lacking. As such, the inclusion of both sexes in clinical as well as preclinical research is of utmost importance. Here, we provide evidence that MIA also sex-dependently influences mitochondrial function in mice, we found that mitochondria in the PFC of females works generally at a higher capacity than those of males. However, after POL exposure the maximal capacity of oxidative phosphorylation – a direct measurement of mitochondrial function – reaches equal values as in females and males. Together with the MIA-induced reduction in the mitochondrial coupling efficiency and reduced transcript expression levels of UCPs 4 and 5 in POL-exposed males, our data suggest that the observed reduction in coupling efficiency is due to an underlying pathological mitochondrial dysfunction. An explicit distinction between physiological uncoupling regulated by uncoupling proteins (UCP) and pathologically defective coupling related to states of mitochondrial dysfunction was demonstrated in earlier studies [55]. Contrary to thermogenic UCPs, the main role of cerebral UCPs is protection against oxidative stress [56, 57]. Along this line, *UCPs 4* and *5* transcript levels are induced by increases in mitochondrial ROS [58] and facilitate the ATPase-independent transport of protons from the intermembrane space into the mitochondrial matrix, thus dissipating the membrane potential and concomitantly reducing ROS production in the ETS [59, 60]. Decreased coupling efficiency and *UCPs 4* and *5* transcripts, as observed in the PFC of MIA males, thus suggest an impairment of defense mechanisms against oxidative stress [61]. Similarly, the reduced expression of both *UCP4* and *UCP5* has been linked with the oxidative-stress-caused loss of dopaminergic neurons in a murine model of Parkinson’s disease [62]. Thus, reduced levels of *UCPs 4* and *5* could be indicative of exacerbated oxidative stress in POL males. If this holds true, antioxidant enzymes like SOD1 and SOD2 would be expected to react to prevent oxidative damage, however, unchanged transcript levels of both enzymes rather suggest a defenseless state of brain cells in POL males against the detrimental effects of ROS. On the contrary, POL females maintain control-like *UCP4*, *UCP5*, and mitochondrial SOD (*SOD2*) transcript expression, suggesting proficient regulation of oxidative stress inside mitochondria. Thus, it seems plausible that the female PFC has a more resilient antioxidant mitochondrial machinery than that of males. Increased transcript expression in antioxidant *SOD1* in females, further suggests a better ROS protection. In agreement with this notion, it has been proposed that the brain of old female rats is better protected against neurodegeneration caused by oxidative stress than that of males because of their higher antioxidant enzymatic activity (mitochondrial glutathione peroxidase) and UCP4 and UCP5 levels [61, 63]. As this link currently remains speculative, future studies should assess the effects of MIA on levels of oxidative stress in females and males, and its consequences on different brain cells in the offspring.

We also found that mitochondrial dynamics are altered in adult offspring after MIA. Mitochondrial dynamics refers to the balance of fission (DRP1-DNM1 and DNM2) and fusion (MFN1, MFN2) events, essential to maintenance of mitochondrial function and optimized bioenergetic capacity [64] keeping the balance of cellular homeostasis, especially under metabolic and environmental stress [51]. Dysfunctional mitochondrial dynamics, leading to abnormal cellular fate, are associated with various diseases, including NDDs, neurodegenerative disorders, metabolic diseases, cardiovascular diseases, and cancers [65]. We observed that both mitochondrial fission and fusion were decreased in PFC of MIA animals, as indexed by reduced levels of *Mfn1, Mfn2, Dnm1, and Dnm2*. We hence would also expect a resulting alteration in ATP production and calcium homeostasis, which may lead to alterations in synaptic release.

We acknowledge limitations in our study. First, our investigation of markers for oxidative stress and fusion and fission is based on RNA expression levels. Arguably, assessing RNA levels has its limitations as it does not provide a complete picture of cellular function, and therefore an extension to protein levels would be desirable to further examine this aspect in future studies. However, the assessment of transcript levels paired with a direct functional assessment in brain tissue allows us to conclude that MIA has the potential to modify mitochondria function.

Second, our study was not designed to assess causal relationships between the mitochondrial and/or molecular changes and behavioral deficits in the present model so that these relationships remain descriptive and speculative at present. Additional studies are warranted to identify the causal relationships between sex-based differences in mitochondrial function, the specific molecular changes in the PFC or AMY (and possibly other brain areas), and the behavioral deficits emerging in MIA-exposed offspring. At the cellular level, estrogens are direct modulators of mitochondrial function [66], thus animal models to identify the mechanism by which estrogens regulate mitochondrial function during GABAergic development, would allow us to understand better these sex-based differences in neurodevelopmental disorders.

In conclusion, we here show that MIA in mice leads long-lasting alterations in mitochondrial functions in the brain of exposed offspring, with the effects displaying certain region- and sex-specificity. Together with previous data [48] our data highlights the potential role of mitochondrial function in MIA-induced neurodevelopmental disorders. Further elucidating the role of mitochondria as a potential mediator of MIA-induced alterations in brain and behavior may not only provide new insight into the pathophysiology of neurodevelopmental disorders with inflammatory etiologies but also offer new therapeutic targets.

### Supplementary information

Figure legends

Figure S1

Figure S2

## Data Availability

Datasets generated during the analysis in the present study are available from the corresponding author on reasonable request.
