# Supplementary material for: Prenatal immune activation in mice induces long-term alterations in brain mitochondrial function
Source: Transl Psychiatry. 2024 Jul 16;14:289. doi: 10.1038/s41398-024-03010-x (PMC11251165; doi:10.1038/s41398-024-03010-x)
Supplement: Supplementary file 1 — Figure legends [file 41398_2024_3010_MOESM1_ESM.docx]

**Supplementary Figure 1. Transcript expression of uncoupling proteins (UCP)s 1 and 2 in PFC and AMY from POL-exposed and control male and female offspring. (a)** Transcript expression analysis of the uncoupling protein 1 (*UCP1*) in the PFC without transcript changes between experimental groups. Multiple comparisons: *P* = 0.1922. **(b)** Transcript expression analysis of the uncoupling protein 2 (*UCP2*) in the PFC without transcript changes between experimental groups. Multiple comparisons: *P* = 0.39. **(c)** Transcript expression analysis of the uncoupling protein 1 (*UCP1*) in the AMY without transcript changes between experimental groups. Multiple comparisons: *P* = 0.4. **(b)** Transcript expression analysis of the uncoupling protein 2 (*UCP2*) in the AMY across experimental groups. Multiple comparisons: *P* = 0.07. Bar plots with individual values represent means ± SD. *n* = 7 per sex from 7 litter per group.

**Supplementary Figure 2.** Representative images of FISH analysis in PFC of control mice. Scale bar: 20 mm. Cytb: channel 1, microglia channel 2, Cox6a1 channel 3, and neurons channel 4.
