# Supplementary figures and images for: Prenatal immune activation in mice induces long-term alterations in brain mitochondrial function
Source: Transl Psychiatry. 2024 Jul 16;14:289. doi: 10.1038/s41398-024-03010-x (PMC11251165; doi:10.1038/s41398-024-03010-x)

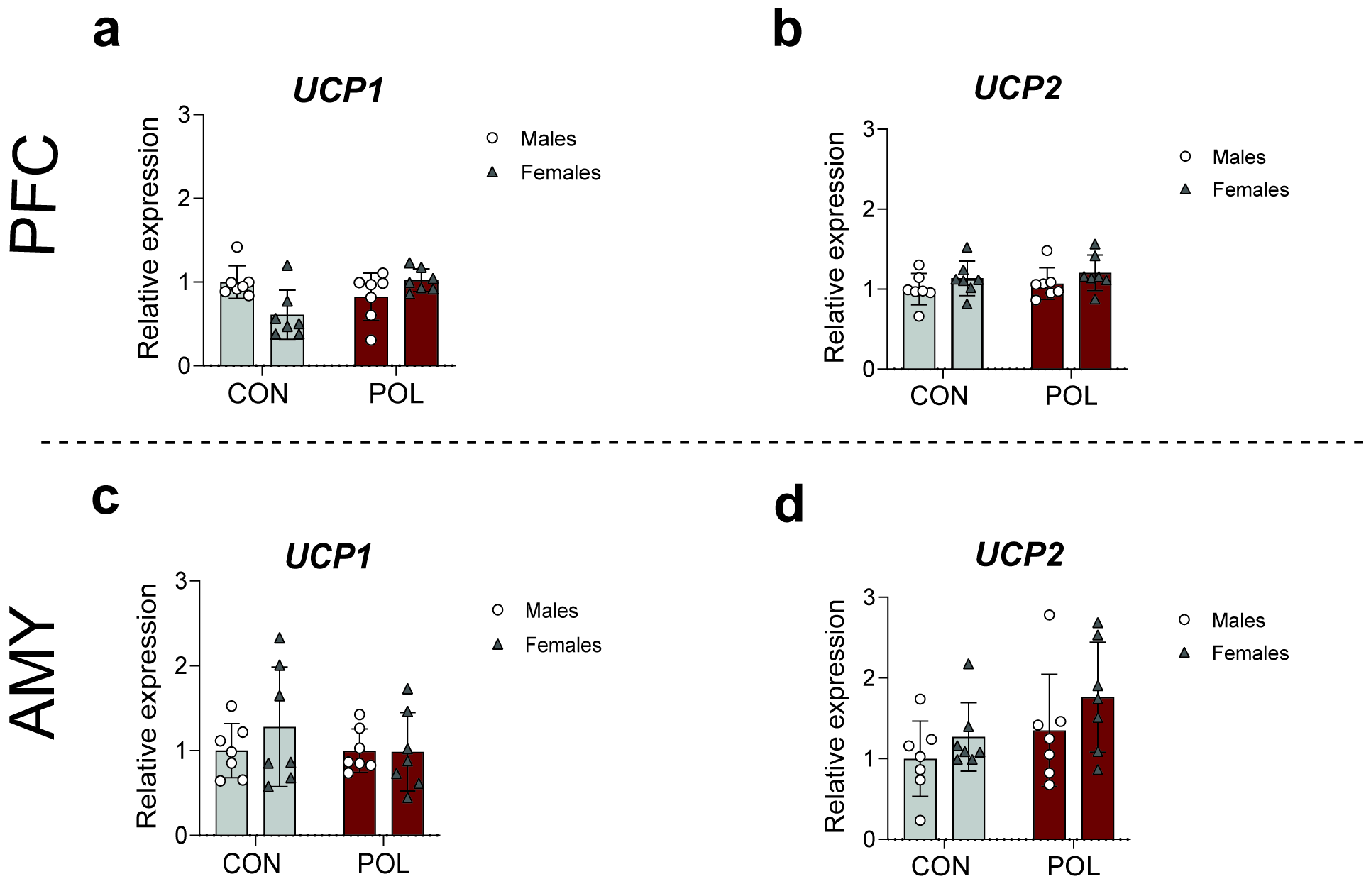

Supplement: Supplementary file 2 — Figure S1 [file 41398_2024_3010_MOESM2_ESM.tif]

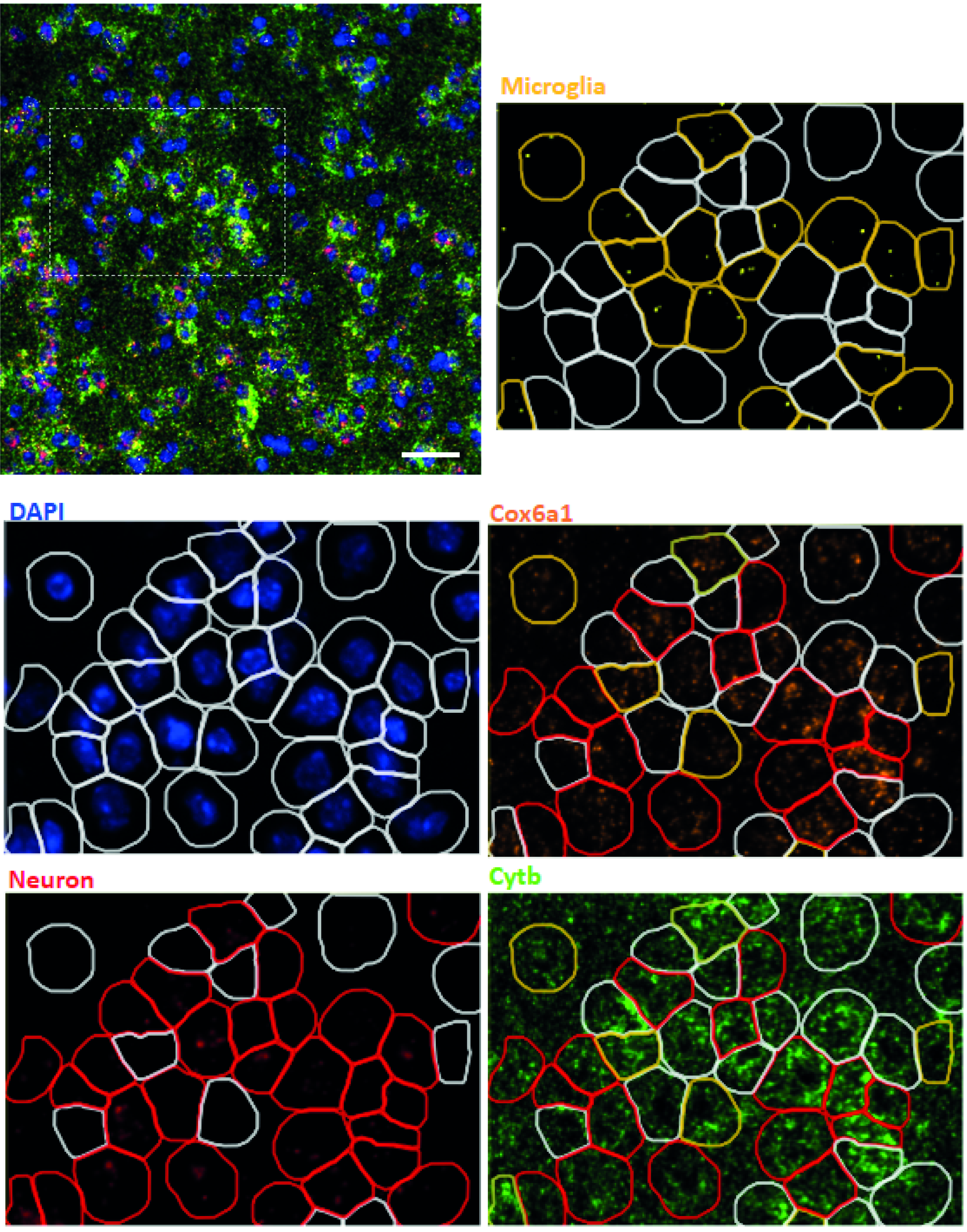

Supplement: Supplementary file 3 — Figure S2 [file 41398_2024_3010_MOESM3_ESM.tif]
